# Serum Inflammatory Factors and Oxidative Stress Factors Are Associated With Increased Risk of Frailty and Cognitive Frailty in Patients With Cerebral Small Vessel Disease

**DOI:** 10.3389/fneur.2021.786277

**Published:** 2022-01-06

**Authors:** Lei Mu, Limin Jiang, Juan Chen, Mei Xiao, Wei Wang, Peipei Liu, Jialing Wu

**Affiliations:** ^1^Clinical College of Neurology, Neurosurgery and Neurorehabilitation, Tianjin Medical University, Tianjin, China; ^2^Department of Geriatrics, Inner Mongolia People's Hospital, Hohhot, China; ^3^Department of Neurorehabilitation and Neurology, Tianjin Huanhu Hospital, Tianjin Key Laboratory of Cerebral Vascular and Neurodegenerative Diseases, Tianjin Neurosurgical Institute, Tianjin, China

**Keywords:** cerebral small vascular disease, cognitive frailty, frailty, CRP, TNF-α, MMP-3, MDA

## Abstract

**Objective:** To study the correlation between serum inflammatory factors, oxidative stress factors and frailty, and cognitive frailty in patients with cerebral small vessel disease (CSVD).

**Methods:** A total of 281 patients with CSVD were selected from Tianjin Huanhu Hospital and Inner Mongolia People's Hospital from March 2019 to March 2021. CSVD was diagnosed by MRI. The FRAIL scale was used to evaluate the frailty of patients. Patients with CSVD with frailty and MMSE score <27 were considered to have cognitive frailty. Patients with non-cognitive frailty were included in the control group. The Montreal Cognitive Assessment (MoCA) and Mini-Mental State Examination (MMSE) were used to evaluate the cognitive function of patients with CSVD. The serum interleukin 6 (IL-6), tumor necrosis factor-alpha (TNF-α), matrix metalloproteinase 3 (MMP-3), superoxide dismutase (SOD), and malondialdehyde (MDA) of patients with CSVD were detected. The correlation between blood inflammatory factors and oxidative stress factors with the frailty and cognitive frailty patients of CSVD were analyzed. Univariate and multivariate logistic regression were used to analyze the correlation between cognitive frailty and CSVD.

**Results:** Among the patients with CSVD selected in this study, female patients and older patients had a higher proportion of frailty (*p* < 0.001). In the Frail group, MoCA score and MMSE score were significantly lower than in the Pre-Frail and Robust groups, Hamilton Depression Scale (HAMD) and Hamilton Anxiety Scale (HAMA) scores were significantly higher than the Pre-Frail and Robust groups, and the differences were statistically significant (*p* < 0.05). Serum CRP, IL-6, TNF-α, MMP-3, and MDA levels in the Frail group were higher, but SOD levels were lower. The levels of serum CRP, IL-6, TNF-α, MMP-3, and MDA in patients with CSVD in the Cognitive Frailty group were significantly higher than those of the Control group, while the levels of SOD were significantly lower than those of the Control group, and the differences were significant (*p* < 0.001). The results of univariate and multivariate logistic regression analysis showed that CRP, TNF-α, MMP-3, and MDA levels were associated with cognitive frailty in patients with CSVD (*p* < 0.05).

**Conclusion:** The increase of serum CRP, TNF-α, MMP-3, and MDA levels are significantly related to the increased risk of frailty and cognitive frailty in patients with CSVD.

## Introduction

Cerebral small vessel disease (CSVD) is a common clinical cerebrovascular disease with a high incidence. Small perforating arterioles, capillaries, and venules of the brain are the most common causes of vascular cognitive disorders and dementia (1–3). With the acceleration of population aging and the high incidence of risk factors for cerebrovascular diseases, CSVD is increasing, which not only increases the social burden but also reduces the quality of life of patients. Therefore, it is of great significance to study the pathogenesis of CSVD and improve the clinical diagnosis and treatment of patients with CSVD.

Frailty is a physiological decline syndrome occurring with aging, which is characterized by reduced functional reserve and increased vulnerability (4). Frail people are less able to adapt to stress factors such as disease or trauma (5). Frailty is closely related to disability, fall, hospitalization, readmission, and death (6). In the process of human aging, all people will have cognitive decline, and some elderly people will eventually suffer cognitive impairment and dementia. Cognitive frailty refers to a clinical syndrome of reduced cognitive reserve (except dementia) combined with physical weakness (7). The specific mechanism of frailty is currently unclear.

In recent years, inflammatory factors have been recognized as risk factors for stroke (8), dementia (9, 10), small vessel disease (11, 12), and frailty (13). Research evidence showed that there was a significant correlation between CSVD and vascular inflammatory factors (14, 15).

The oxidative stress pathway plays an important role in the frailty and cognitive impairment of patients with CSVD. Oxidative stress can cause lipid oxidative damage, while malondialdehyde (MDA) is a common end product of lipid peroxidation, and increased MDA levels are generally considered to be a sign of oxidative stress pathway activation. Therefore, MDA can indirectly reflect the metabolism of oxygen free radicals in the body (16, 17). Superoxide dismutase (SOD) is an important antioxidant enzyme with strong oxidizing properties in the human body, which can effectively eliminate excess oxygen free radicals and derivatives of oxygen free radicals, and protect cells from damage (18). The specific mechanism of oxidative stress in the frailty and cognitive dysfunction of patients with CSVD is still unclear.

Therefore, inflammatory factors and oxidative stress factors may be one of the possible mechanisms of frailty and cognitive frailty. However, the pathogenesis of frailty and cognitive frailty in patients with CSVD is still unclear. This study aimed to study the effects of inflammation and oxidative stress on frailty and cognitive frailty in patients with CSVD and to provide evidence for intervention frailty and cognitive frailty.

## Methods

### Patients

A total of 281 patients with CSVD (including lacunar infarction, high white matter signal, microhemorrhage, perivascular space, and brain atrophy) diagnosed by head MRI in Tianjin Huanhu Hospital and Inner Mongolia People's Hospital from March 2019 to March 2021 were collected, aged 37–90 years old, with an average of 65.95 ± 9.07 years old. The inclusion criteria were (1) stable health status, able to move independently, able to understand and correctly answer questions, and assist in completing questionnaires; and (2) informed consent and voluntary participation in the study. The exclusion criteria were (1) patients with severe physical dysfunction, unable to cooperate with the completion of the evaluation, and accompanying visitors; (2) people with speech or hearing impairment; (3) severe liver and kidney diseases, vital organ failure, and expected survival of <1 year; (4) acute infection; (5) those with contraindications to magnetic resonance examination; (6) cognitive frailty caused by factors such as Alzheimer's disease, Parkinson's disease, sequelae of cerebral infarction, and severe demyelinating disease; (7) those who have taken nootropic drugs for a long time in the past; and (8) those who have communication difficulties and are unable to complete the neuropsychological assessment.

### FRAIL Scale

The FRAIL scale consists of five self-reported components. (1) Fatigue: in the past 4 weeks, have you often felt tired? (2) Resistance: if you do not have a rest or do not have mobility aids to climb 10 stairs, do you find it difficult? (3) Ambulation: do you find it difficult to walk 500~600 m without the assistance of mobility aids? (4) Illnesses: do you have five or more of the following diseases: hypertension, diabetes, acute heart attack, stroke, malignant tumors, congestive heart failure, asthma, arthritis, chronic lung disease, kidney disease, and angina pectoris? (5) Loss of weight: in the past 1 year or less, has your body weight decreased by ≥ 5%? (19). Patients having three or more of the above can be diagnosed as Frail, meeting one or two of the above can be diagnosed as Pre-Frail, and those who do not meet any of the above are diagnosed as Robust (20, 21).

### Assessment of Cognitive Function

The Montreal Cognitive Assessment Scale (MoCA) (22) and Mini-mental State Examination (MMSE) were used to assess the cognitive function of patients with CSVD (23).

The Montreal Cognitive Assessment Scale includes 10 subtests, including visuospatial/executive abilities, naming, attention to digits, attention to letters, attention to subtraction, language repetition, language fluency, abstraction, delayed recall, and orientation. The total score is 30 points. The MoCA score needs to refer to the educated years of the evaluator. If the educated period of the evaluator is ≤ 12 years, one point should be added to the measured score to correct the bias caused by the level of education.

The total score of MMSE is 30. A score of 27–30 indicates normal cognitive function, a score of <27 indicates cognitive dysfunction.

In this study, patients with CSVD with frailty and an MMSE score <27 were considered to have cognitive frailty (23).

### Emotional Assessment

The Hamilton Depression Scale (HAMD) (24) and Hamilton Anxiety Scale (HAMA) (25) were used to evaluate the mood of the patient.

### Laboratory Index Testing

All patients fasted for 12 h in the morning and then 10 ml of peripheral venous blood was drawn. Serum inflammatory factors and oxidative stress factors, such as C-reactive protein (CRP), interleukin-6 (IL-6), tumor necrosis factor α (TNF-α), matrix metalloproteinase-3 (MMP-3), superoxide dismutase (SOD), and malondialdehyde (MDA) were detected by using ELISA.

### Statistical Analysis

The data were analyzed by SPSS (version 22.0, Chicago, IL, USA), measurement data were presented as mean ± SD, ANOVA, *t*-test was used for statistical analysis between groups, and χ^2^-test was used for categorical variables [*n* (%)]. The frailty was used as the dependent variable, and the clinical data and the level of inflammatory factors and oxidative stress factors were used as independent variables to conduct a univariate regression analysis. Cognitively frailty was used as the dependent variable, and the statistically different indicators of univariate regression analysis were used as independent variables to perform multivariate logistic regression analysis. Univariate and multivariate logistic regressions were used to analyze the association between cognitive frailty with CSVD. All test results were two-tailed, and *p* < 0.05 indicated that the difference was statistically significant.

## Results

### FRAIL and Demographic Data

The correlation between FRAIL score and gender, age, BMI, hypertension, smoking, and drinking is shown in Table 1. The analysis results showed that among the patients with CSVD selected in this study, female patients and older patients had a higher proportion of frailty (*p* < 0.001). There was no significant correlation between BMI, hypertension, smoking, drinking, and frailty (*p* > 0.05).

**Table 1:** Comparison of demographic information between the Frail, Pre-Frail, and Robust groups.

	**Frail**	**Pre-Frail**	**Robust**	***p***
	**(***n =*** 32)**	**(***n =*** 112)**	**(***n =*** 137)**	
Gender [*n* (%)]				<0.001
Male	11 (34.38%)	78 (69.64%)	109 (79.56%)	
Female	21 (65.63%)	34 (30.36%)	28 (20.44%)	
Age (years)^a^	74.78 ± 9.62	67.91 ± 7.47	62.28 ± 8.23	<0.001
BMI (kg/m^2^)^a^	24.59 ± 3.35	24.71 ± 2.96	25.73 ± 2.75	0.38
Hypertension [*n* (%)]				0.43
Yes	27 (84.38%)	99 (88.39%)	113 (82.48%)	
No	5 (15.63%)	13 (11.61%)	24 (17.52%)	
Smoking [*n* (%)]				0.41
Yes	11 (34.38%)	45 (40.18%)	63 (45.99%)	
No	21 (65.63%)	67 (59.82%)	74 (54.01%)	
Drinking [*n* (%)]				0.07
Yes	4 (12.50%)	31 (27.68%)	45 (32.85%)	
No	28 (87.50%)	81 (72.32%)	92 (67.15%)	

*BMI, body mass index*.

a *Data are expressed as mean ± SD*.

### Correlation Between Frailty and MoCA, MMSE, HAMD, and HAMA Scales

The correlation between frailty and MoCA and MMSE scores is shown in Table 2. In the Frail group, MoCA and MMSE scores were significantly lower than the Pre-Frail and Robust groups, HAMD and HAMA scores were significantly higher than the Pre-Frail and Robust groups, and the differences were statistically significant (*p* < 0.05).

**Table 2:** Comparison of Montreal Cognitive Assessment Scale (MoCA), Mini-mental State Examination (MMSE), Hamilton Depression Scale (HAMD), and Hamilton Anxiety Scale (HAMA) scores among the Frail, Pre-Frail, and Robust groups.

	**Frail**	**Pre-Frail**	**Robust**	***p***
	**(***n =*** 32)**	**(***n =*** 112)**	**(***n =*** 137)**	
MoCA^a^	18.16 ± 4.00	20.32 ± 4.03	22.96 ± 3.62	<0.001
MMSE^a^	22.84 ± 3.71	24.82 ± 3.07	26.55 ± 2.35	<0.001
HAMD^a^	6.16 ± 3.14	3.72 ± 2.72	2.39 ± 2.12	<0.001
HAMA^a^	6.38 ± 2.84	3.79 ± 2.46	2.58 ± 1.83	<0.001

a *Data are expressed as mean ± SD*.

### Frailty Was Correlated With Levels of Inflammatory Factors and Oxidative Stress Factors

**Figure 1:**
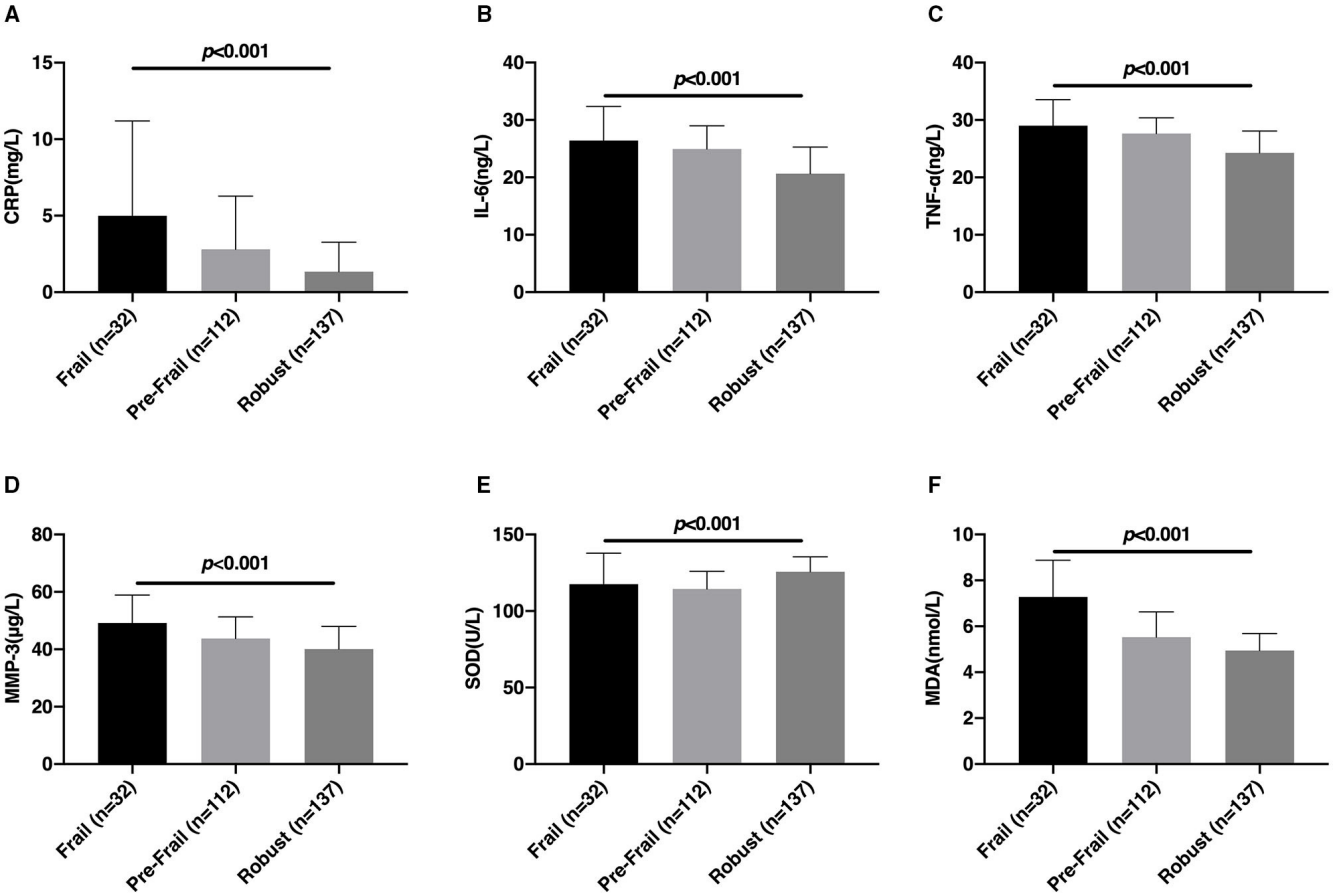
Comparison of the levels of inflammatory factors and oxidative stress factors among the Frail, Pre-Frail, and Robust groups. The comparison of serum CRP **(A)**, IL-6 **(B)**, TNF-α **(C)**, MMP-3 **(D)**, SOD **(E)**, and MDA **(F)** levels between the Frail, Pre-Frail, and Robust groups. CRP, C-reactive protein; IL-6, Interleukin-6; TNF-α, tumor necrosis factor α; MMP-3, matrix metalloproteinase-3; SOD, superoxide dismutase; MDA, malondialdehyde.

The comparison results of the inflammatory factors and oxidative stress factors between the Frail, Pre-Frail, and Robust groups are shown in Figure 1. The CRP, IL-6, TNF-α, MMP-3, SOD, and MDA levels of patients with CSVD between Frail, Pre-Frail, and Robust groups are significantly different (*p* < 0.05). Serum CRP, IL-6, TNF-α, MMP-3, and MDA levels in the Frail group were higher, but SOD levels were lower.

### Cognitive Frailty and Demographic Data of Patients With CSVD

The demographic data of patients with CSVD in the Cognitive Frailty and Non-Cognitive Frailty (Control) groups are shown in Table 3. In the Cognitive Frailty group, the number of women in patients with CSVD and their age were significantly higher than those in the Control group, and the difference was statistically significant (*p* < 0.001), there was no statistically significant difference between the BMI, hypertension, smoking, and drinking for patients with CSVD in the Cognitive Frailty and Control groups (*p* > 0.05).

**Table 3:** Comparison of demographic data of patients with cerebral small vessel disease (CSVD) in the Cognitive Frailty and Control groups.

	**Cognitive frailty**	**Control**	***p***
	**(***n =*** 26)**	**(***n =*** 255)**	
Gender [*n* (%)]			<0.001
Male	9 (34.62%)	189 (74.12%)	
Female	17 (65.38%)	66 (25.88%)	
Age (years)^a^	76.00 ± 9.64	64.93 ± 8.38	<0.001
BMI (kg/m^2^)^a^	24.48 ± 2.99	25.27 ± 2.93	0.19
Hypertension [*n* (%)]			0.52
Yes	21 (80.77%)	218 (85.49%)	
No	5 (19.23%)	37 (14.51%)	
Smoking [*n* (%)]			0.40
Yes	9 (34.62%)	110 (43.14%)	
No	17 (65.38%)	145 (56.86%)	
Drinking [*n* (%)]			0.12
Yes	4 (15.38%)	76 (29.80%)	
No	22 (84.62%)	179 (70.20%)	

*BMI, body mass index*.

a *Data are expressed as mean ± SD*.

### Correlation Between Cognitive Frailty and HAMD and HAMA Scores in Patients With CSVD

The correlation between cognitive frailty and HAMD and HAMA scores in the patients with CSVD is shown in Table 4. In the Cognitive Frailty group, the HAMD and HAMA scores of patients with CSVD were significantly higher than those of the Control group, and the differences were statistically significant (*p* < 0.001).

**Table 4:** Comparison of HAMD and HAMA scores between Cognitive Frailty and Control groups.

	**Cognitive frailty** **(***n =*** 26)**	**Control** **(***n =*** 255)**	***p***
HAMD^a^	6.04 ± 3.32	3.07 ± 2.55	<0.001
HAMA^a^	6.31 ± 3.08	3.21 ± 2.26	<0.001

*HAMD, Hamilton Depression Scale; HAMA, Hamilton anxiety scale*.

a *Data are expressed as mean ± SD*.

### Correlation Between Cognitive Frailty and the Levels of Inflammatory Factors and Oxidative Stress Factors in Patients With CSVD

**Figure 2:**
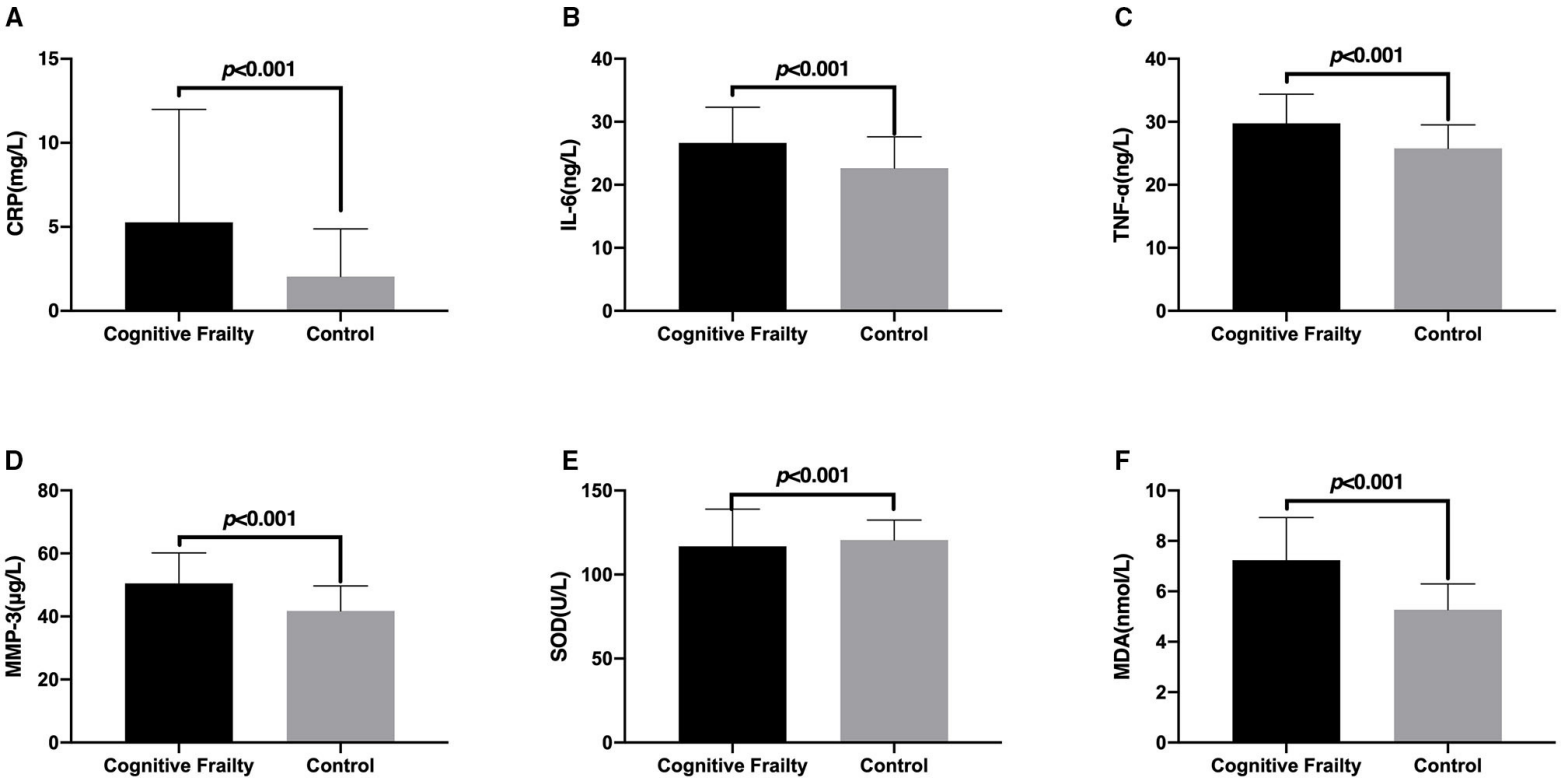
Comparison of the levels of inflammatory factors and oxidative stress factors in patients with cerebral small vessel disease (CSVD) between the Cognitive Frailty and Control groups. The comparison of serum CRP **(A)**, IL-6 **(B)**, TNF-α **(C)**, MMP-3 **(D)**, SOD **(E)**, and MDA **(F)** levels of patients with CSVD between the Cognitive Frailty and Control groups. CRP, C-reactive protein; IL-6, Interleukin-6; TNF-α, tumor necrosis factor α; MMP-3, matrix metalloproteinase-3; SOD, superoxide dismutase; MDA, malondialdehyde.

The correlation between cognitive frailty and CRP, IL-6, TNF-α, MMP-3, SOD, and MDA in patients with CSVD is shown in Figure 2. The levels of serum CRP, IL-6, TNF-α, MMP-3, and MDA in patients with CSVD with cognitive frailty were significantly higher than those of the Control group, while the level of SOD was significantly lower than those of the Control group, and the differences were extremely significant (*p* < 0.001).

### Univariate Logistic Regression Analysis

We performed a univariate logistic regression analysis of the subjects' demographic data, serum inflammatory factors, and oxidative stress factors, and the results are shown in Table 5. Univariate analysis showed that gender, age, BMI, CRP, IL-6, TNF-α, MMP-3, and MDA were all associated with cognitive frailty in patients with CSVD (*p* < 0.05). There was no significant correlation between hypertension, smoking, drinking, SOD, and the risk of cognitive frailty in patients with CSVD (*p* > 0.05).

**Table 5:** Univariate logistic regression analysis results of cognitive frailty in patients with CSVD.

	**B**	**SE**	**Wald**	**Sig**.	**Exp(B)**	**95% CI**	
						**Lower**	**Upper**
Female	1.69	0.44	14.97	<0.01	5.41	2.30	12.72
Age	−2.17	1.03	4.43	0.04	0.12	0.02	0.86
BMI	1.18	0.43	7.74	0.01	3.26	1.42	7.49
Hypertension	0.34	0.53	0.41	0.52	1.40	0.50	3.95
Smoking	0.36	0.43	0.70	0.40	1.43	0.62	3.34
Drinking	0.85	0.56	2.29	0.13	2.34	0.78	7.01
CRP	0.15	0.04	13.53	<0.01	1.16	1.07	1.26
IL-6	0.15	0.04	13.22	<0.01	1.16	1.07	1.26
TNF-α	0.27	0.06	21.09	<0.01	1.31	1.17	1.47
MMP-3	0.12	0.03	21.53	<0.01	1.13	1.07	1.19
SOD	−0.01	0.02	0.06	0.81	1.00	0.96	1.04
MDA	0.99	0.16	37.16	<0.01	2.68	1.95	3.69

*The adjusted R^2^-values of regression analysis for gender, age, BMI, hypertension, smoking, drinking, CRP, IL-6, TNF-α, MMP-3, SOD, and MDA were 0.060, 0.019, 0.026, 0.001, 0.002, 0.005, 0.068, 0.048, 0.081, 0.086, 0.004, and 0.211, respectively. BMI, body mass index; CRP, C-reactive protein; IL-6, Interleukin-6; TNF-α, tumor necrosis factor α; MMP-3, matrix metalloproteinase-3; SOD, superoxide dismutase; MDA, malondialdehyde; SE, standard error; CI, confidence interval*.

### Multivariate Logistic Regression Analysis

We used multivariate logistic regression to analyze the correlation between cognitive frailty and CSVD, and adjusted the demographic data of the patients with CSVD. The analysis results are shown in Table 6. The results showed that CRP, TNF-α, MMP-3, and MDA levels were associated with cognitive frailty in patients with CSVD (*p* < 0.05).

**Table 6:** Multivariate logistic regression analysis results of cognitive frailty in patients with CSVD.

	**B**	**SE**	**Wald**	**Sig**.	**Exp(B)**	**95% CI**	
						**Lower**	**Upper**
CRP	0.99	0.45	4.82	0.03	2.69	1.11	6.49
IL-6	0.81	0.52	2.49	0.12	2.26	0.82	6.21
TNF-α	1.57	0.59	7.14	0.01	4.81	1.52	15.23
MMP-3	1.52	0.58	6.85	0.01	4.56	1.46	14.19
SOD	−0.38	0.45	0.71	0.40	0.69	0.29	1.65
MDA	2.15	0.65	10.87	0.00	8.61	2.39	30.95

*R^2^ = 0.452. BMI, body mass index; CRP, C-reactive protein; IL-6, Interleukin-6; TNF-α, tumor necrosis factor α; MMP-3, matrix metalloproteinase-3; SOD, superoxide dismutase; MDA, malondialdehyde; SE, standard error; CI, confidence interval*.

## Discussion

According to the results of this study, women and older people were at greater risk of frailty and cognitive frailty, which was consistent with the findings of Kang et al. (26). Kim et al. (27) found that age and gender were important factors affecting frailty. Women were more likely to have frailty symptoms than men, which may be related to female physiological characteristics. Elderly patients are more prone to frailty symptoms due to decreased physical function and more complicated diseases. Solfrizzi et al. (28) found that with the increase of age, the incidence of cognitive frailty in elderly women was higher than that in elderly men. According to our research results, frailty and cognitive frailty patients were more likely to have emotional disorders, these symptoms may be a challenge to the patient's normal life. Therefore, it can be considered that improving the frailty and cognitive frailty symptoms of patients with CSVD has important clinical significance.

The results of this study showed that compared with the Pre-Frail and Robust groups, the serum CRP, IL-6, TNF-α, MMP-3, and MDA levels of patients with CSVD in the Frail group were higher, and the SOD level was lower. The study found that in patients with mild cognitive frailty and mild to moderate Alzheimer's disease, the pro-inflammatory factor TNF-α was associated with an increased risk of physical frailty (29). Similarly, studies have shown that TNF-α is associated with the frailty phenotype (30). In a longitudinal study, IL-6 was found to be associated with frailty (31). Another study found that IL-6 levels increased with age and were negatively correlated with exercise endurance in frail elderly people (32). With age, the levels of serum IL-6, CRP, and TNF-a increase significantly, which were positively correlated with the decline of muscle mass, strength, and function, and promoted the occurrence of frailty (33). We know that inflammatory factors, such as IL-6, CRP, and TNF-α can directly or indirectly affect motor function, endocrine function, circulatory function, and neurological function of patients with CSVD and further participate in the occurrence, progression, and outcome of frailty. Ingles et al. (34) found that MAD, a product of plasma lipid peroxidation, was increased in the Frailty group of elderly people in the community, which proved to be related to frailty.

After using multivariate logistic regression analysis and adjusting the demographic data, we found that CRP, TNF-α, MMP-3, and MDA were associated with cognitive frailty in patients with CSVD. Studies have found that the use of dexmedetomidine can reduce IL-6 levels, improve long-term cognitive dysfunction and anti-inflammatory effects (35). In addition, in a rat model, researchers found that dexmedetomidine can reduce the expression levels of TNF-α and NF-κB and improve postoperative cognitive function in rats (36). Another study showed that MMP-3 may be involved in the early pathogenesis of Alzheimer's disease, which may lead to neuronal degeneration, neurofibrillary tangles, and cognitive dysfunction (37). MDA is an independent risk factor for postoperative cognitive dysfunction in elderly patients undergoing hip fracture surgery (38). The results of a study showed that luteolin can prevent the increase of TNF-α, IL-1β, and IL-6, and MDA, increase the activity of SOD and glutathione peroxidase, inhibit the excessive activation of microglia and the proliferation of astrocytes (especially in the hippocampus and cortex), and improve learning and learning ability, which is expected to improve the cognitive dysfunction of patients (39). The above evidence shows that CRP, TNF-α, MMP-3, and MDA are expected to become targets for improving the cognitive function of patients with CSVD, and have potential clinical therapeutic value. Although IL-6 and SOD levels were not found in this study to be associated with the risk of cognitive frailty in patients with CSVD, we speculate that the reason may be related to the small sample size of patients with CSVD with cognitive frailty, therefore, we plan to further expand the sample size for research in the later period.

Studies have found that inflammation in the brain can promote the release of peripheral blood inflammatory factors, and these factors can change the permeability of the blood-brain barrier, thereby promoting the entry of inflammatory factors into the brain (40, 41). After inflammatory factors enter the brain, they can induce brain-related diseases. For example, inflammatory factors play a key role in the occurrence of Alzheimer's disease (AD), which has been supported by research evidence (42, 43). In addition, the latest research evidence showed that patients who had recovered from COVID-19 had cognitive dysfunction, which may be related to the concentration of CRP inflammatory factors (44). Researchers have also found that intravenous injection of dexmedetomidine can reduce serum inflammatory factors and improve the cognitive function of patients undergoing tonsillectomy (45).

In addition, this study found that the MoCA score and MMSE score of the Cognitive Frailty group were significantly lower than those of the Control group, suggesting that cognitive frailty in patients with CSVD is closely related to the occurrence of cognitive dysfunction, but its internal linkage mechanism needs further study.

We know that the causes of frailty and cognitive frailty are very complex (46, 47). Taken together, they involve the interaction of genetic, physiological, psychological, social, and environmental factors (48). A number of studies have shown the relationship between inflammation, oxidative stress, frailty, and cognitive frailty (49–51). These studies suggest that we can establish a link between neurodegenerative diseases and oxidative stress. However, relevant research is still lacking, especially to assess whether elderly frail patients or patients with CSVD can effectively improve disease progression and slow cognitive decline by increasing the intake of antioxidants or anti-inflammatory agents. It can be inferred from this study that reducing inflammation and oxidative stress may delay the progression of patients with CSVD, but further clinical trials and in-depth studies are needed for drug treatments to inhibit inflammation and oxidative stress. In addition, based on the current lack of understanding of cognitive weakness, the treatment of elderly patients with neurodegenerative diseases still faces considerable challenges to overcome (52).

The sample size of this study is limited, which can be expanded in future studies. At the same time, this study is a cross-sectional study, without tracking the health status of patients, especially the adverse outcomes, such as death. In future research, multidisciplinary team cooperation will be adopted to provide personalized guidance for patients with small cerebral vascular disease and cognitive weakness and a multidisciplinary combination of frailty prevention and management.

## Conclusion

In summary, our research results showed that the increase in serum CRP, TNF-α, MMP-3, and MDA levels are associated with an increased risk of cognitive frailty in patients with CSVD. At present, there is no unified treatment method for CSVD. At the same time, the treatment of frailty and cognitive frailty is not clear. Studies have shown that anti-inflammatory treatment may be helpful. The clinical significance of this study is to explore the relationship between patients with CSVD with combined frailty, cognitive frailty, and inflammatory factors, and oxidative stress factors, to provide new ideas for improving the cognitive frailty of patients with CSVD.

## Data Availability Statement

The original contributions presented in the study are included in the article/supplementary material, further inquiries can be directed to the corresponding author/s.

## Ethics Statement

The studies involving human participants were reviewed and approved by the Ethics Committee of the Tianjin Huanhu Hospital (No. 2019-29). The patients/participants provided their written informed consent to participate in this study.

## Author Contributions

JW contributed to the experiment design, manuscript draft, and data analysis. LM contributed to the experiment implementation and manuscript draft. LJ, JC, MX, WW, and PL analyzed the data. All authors read and approved the final manuscript.

## Conflict of Interest

The authors declare that the research was conducted in the absence of any commercial or financial relationships that could be construed as a potential conflict of interest.

## Publisher's Note

All claims expressed in this article are solely those of the authors and do not necessarily represent those of their affiliated organizations, or those of the publisher, the editors and the reviewers. Any product that may be evaluated in this article, or claim that may be made by its manufacturer, is not guaranteed or endorsed by the publisher.
